# Evaluation of Posture-Dependent Signal Intensity and Contrast Alterations in Low-Field Brain Magnetic Resonance Imaging

**DOI:** 10.3390/diagnostics16091333

**Published:** 2026-04-29

**Authors:** Chang-Soo Yun, Changheun Oh, Kyuseok Kim, Seong-Hyeon Kang, Hajin Kim, Youngjin Lee, Jun-Young Chung, Gun Choi

**Affiliations:** 1 Neuroscience Research Institute, Gachon University Gil Medical Center, 24, Namdong-daero 774beon-gil, Namdong-gu, Incheon 21565, Republic of Korea; csyun0709@gmail.com; 2 Department of Software and Communications Engineering, Hongik University, 2639, Sejong-ro, Jochiwon-eup, Sejong-si 30016, Republic of Korea; choh@hongik.ac.kr; 3 Institute of Human Convergence Health Science, Gachon University, 191, Hambakmoe-ro, Yeonsu-gu, Incheon 21936, Republic of Korea; kskim502@gachon.ac.kr; 4 Department of Radiological Science, Gachon University, 191, Hambakmoe-ro, Yeonsu-gu, Incheon 21936, Republic of Korea; tjdgus7345@gachon.ac.kr; 5 Department of Health Science, General Graduate School of Gachon University, 191, Hambakmoe-ro, Yeonsu-gu, Incheon 21936, Republic of Korea; happida3@gachon.ac.kr; 6 Department of Neuroscience, College of Medicine, Gachon University, 38-13, Dokjeom-ro 3beon-gil, Namdong-gu, Incheon 21565, Republic of Korea; 7 Department of Neurosurgery, Pohang Woori Hospital, 256, Posco-daero, Buk-gu, Pohang-si 37755, Gyeongsangbuk-do, Republic of Korea; spine.choi@gmail.com

**Keywords:** low-field magnetic resonance imaging, posture-dependent signal, signal-to-noise ratio, contrast-to-noise ratio, signal intensity ratio, gray-level co-occurrence matrix, intensity

## Abstract

**Background/Objectives**: Most brain magnetic resonance imaging (MRI) is performed in supine position, although posture may influence cerebrovascular signal characteristics through gravity-related physiological changes. However, posture-dependent vascular signal alterations on low-field MRI have not been sufficiently quantified. This study aimed to quantify posture-related internal carotid artery (ICA) signal alterations using low-field MRI by comparing seated and supine images with intensity-, noise-, and texture-based metrics. **Methods**: Nine healthy adults (20–69 years old; one female) underwent 0.25 T tilting MRI in supine and seated postures. 3D gradient echo T1-weighted images were obtained. The bilateral ICA regions of interest (ROI) and adjacent reference ROI were manually delineated. The signal-to-noise ratio (SNR), contrast-to-noise ratio (CNR), signal intensity ratio (SIR), gray-level co-occurrence matrix (GLCM) texture features (contrast, correlation, energy, and homogeneity) were extracted and compared between postures using Wilcoxon signed-rank tests. **Results**: Seated posture produced significantly higher ICA signal intensity metrics than the supine posture, with increased SNR (median 17.11 vs. 13.48), CNR (median 21.94 vs. 18.36), and SIR (median 10.84 vs. 9.54) (*p* = 0.004). GLCM texture analysis demonstrated a significant decrease in contrast in the seated position (median 62.01 vs. 145.92; *p* = 0.004), whereas correlation, energy, and homogeneity showed no significant between-posture differences. **Conclusions**: Low-field MRI was sensitive to posture-dependent ICA signal alterations. ICA-based metrics may provide quantitative markers of gravity-related cerebrovascular adaptation.

## 1. Introduction

Postural changes in the human body modify the distribution of blood and cerebrospinal fluid (CSF) owing to gravity, leading to adaptive physiological responses in the cerebrovascular system [1,2,3]. In the seated positions, gravity causes blood to pool in the lower parts of the body, whereas transitioning to a supine position redistributes blood toward the central circulation, resulting in changes in cardiac output, arterial blood pressure, and autoregulation [4,5]. In particular, posture-induced changes in the hydrostatic pressure gradient and redistribution of intravascular pressure influence both the structural and functional properties of blood vessels, thereby activating the physiological mechanisms involved in the regulation of cerebral blood flow [2,5,6]. Seated postures dominate daily human activities and reflect a physiological baseline in which gravity persistently influences the cerebrovascular system.

The arteries play a central role in actively regulating cerebral blood flow under changing hemodynamic conditions; thus, a precise assessment of arterial responses to postural changes in required [6,7,8]. Posture-induced resetting of hydrostatic pressure gradients across the brain elicits stretch-sensitive responses in arterial smooth muscles, leading to vasoconstriction or vasodilation and activation of the myogenic response that maintains cerebral perfusion pressure [9,10,11,12]. Among these arteries, the internal carotid artery (ICA) is the principal conduit for cerebral arterial inflow, and owing to its anatomical orientation, is particularly susceptible to gravity-related changes in hydrostatic pressure and intravascular pressure distribution [1,13]. The ICA courses vertically along the neck, and posture-related alterations in the hydrostatic pressure gradient exert substantial effects on this vessel, resulting in measurable changes in vessel diameter, blood flow velocity, and pulsatile properties [14]. Importantly, posture-dependent symptoms, such as dizziness, syncope, headache, and cognitive dysfunction, are often absent or unremarkable in the supine position, thereby constraining the capacity of conventional imaging modalities to explain their pathophysiological mechanisms [15,16,17]. Such posture-dependent symptoms may reflect functional impairment in ICA blood inflow and pressure regulation under gravitational conditions rather than intrinsic structural abnormalities of the cerebral tissue [18,19,20]. Thus, posture-specific quantification of ICA blood flow and signal properties provides an important noninvasive approach for understanding gravity-dependent cerebrovascular regulation.

Considering that gravity-related postural changes exert complex effects on the ICA, medical imaging approaches capable of noninvasive quantification and visualization of these physiological changes are required. Magnetic resonance imaging (MRI) is a noninvasive method for visualizing vascular anatomy and evaluating physiological blood flow changes using techniques such as arterial spin labeling and phase-contrast MRI [21,22,23]. However, conventional MRI approaches frequently require prolonged scanning times and intricate protocols. Conversely, gradient echo-based MRI leverages sensitivity to blood T1 shortening and inflow effects, providing efficient visualization of vascular anatomy and signal alterations within short acquisition times [24].

Most cerebrovascular imaging studies using conventional MRI have been conducted primarily in the supine position, which limits their ability to fully observe the gravity-dependent hemodynamic changes that occur under real-life physiological conditions [25]. Also, the majority of conventional MRI systems operate at high magnetic field strengths (≥1.5 T), requiring helium-based cryogenic cooling to maintain super conductivity. This requirement results in increased system size and weight, imposing substantial structural constraints on the scanner design. In addition, the fixed-magnet configuration and enclosed-bore environment of high-field MRI systems preclude scanner mobility and restrict image acquisition in the supine position. Low-field MRI systems operating at field strengths below 1.0 T have recently attracted attention as promising tools for evaluating gravity-related postural effects [3]. Low-field MRI systems are based on lightweight magnets and open configurations, eliminating the need for superconducting cooling infrastructure. These characteristics afford greater flexibility in system design and enable image acquisition while maintaining the subjects in seated postures [15]. Such low-field MRI system features allow the direct observation of physiological states under postures in which gravitational forces are actively exerted in daily life. However, posture-related studies using low-field MRI have predominantly focused on CSF hydrodynamics, including circulation, flow velocity, and volumetric changes [15,26,27]. In contrast, quantitative assessment of vascular signals remains relatively limited. Although several studies have suggested that gravitational loading associated with postural changes may induce physiological alterations in the carotid arterial system, systematic evaluations of how these changes are reflected in MRI-derived vascular signal intensity, contrast, and spatial signal distribution under seated conditions remain scarce.

This study aimed to investigate gravity-related signal alterations in the ICA using low-field MRI by comparing seated and supine postures with quantitative intensity-, contrast-, and texture-based imaging metrics. While signal intensity serves as a fundamental metric reflecting absolute signal changes associated with arterial inflow effects, signal-to-noise ratio (SNR) analysis was additionally incorporated to assess the robustness of the observed ICA signal alterations [28,29]. Furthermore, contrast-to-noise ratio (CNR) analysis was employed to evaluate posture-dependent changes in the relative visibility of the ICA with respect to surrounding tissues, and gray-level co-occurrence matrix (GLCM)-based texture analysis was applied to characterize spatial pattern alterations in arterial signals. Changes in gravitational loading may influence not only blood flow velocity but also flow patterns and the spatial distribution of intravascular spins, which may manifest as alterations in pixel-wise correlations and signal heterogeneity within low-field MRI images [30].

## 2. Materials and Methods

### 2.1. MRI Data Acquisition

**Figure 1:**
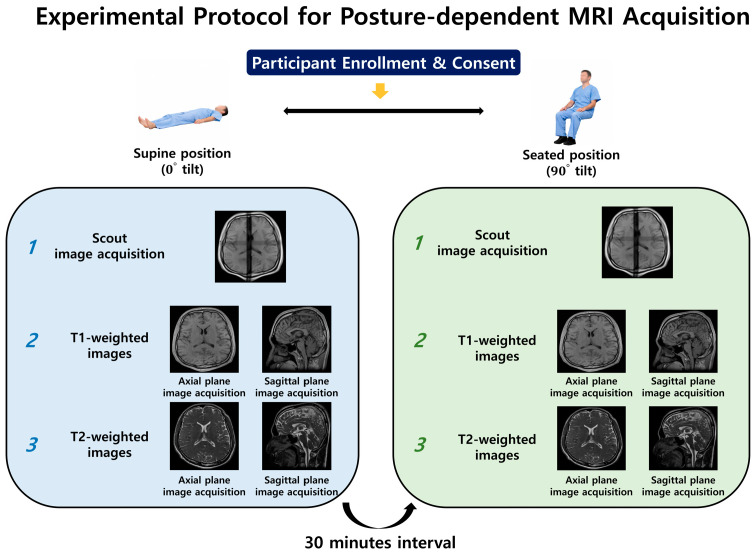
Experimental workflow showing MRI acquisitions across supine and seated postural conditions.

This study was approved by the Institutional Review Board of the Gachon University Gil Medical Center (IRB No. GCIRB2024-209). The participants were healthy adult men and women aged 20–69 years. Individuals were excluded if they had implanted medical devices such as pacemakers, implantable cardioverter-defibrillators or probes, intracranial vascular clips, orthopedic clips, pins, screws, other metallic implants, ocular implants resulting from eye surgery, intrauterine devices, or a history of neurological disease. All participants were informed of the experimental protocol in advance and written informed consent was obtained prior to enrollment. Total nine participants were involved in this study to compare the gravity effect between supine and seated position (age = 31.44 ± 14.06 years, one female and eight males). All MRI images were obtained using a 0.25 T tilting MRI system (G-Scan Brio, Esaote, Genoa, Italy) with a 4-channel brain coil, which allowed image acquisition in both supine and seated positions under the influence of gravity. Each participant underwent MRI in two postural conditions: conventional supine and seated positions. Prior to the MRI image acquisition, a two-dimensional scout image was obtained using a spin-echo sequence to determine the anatomical position. Scout images were used to ensure consistent head alignment and imaging coverage across all acquisitions. Second, T1-weighted images were acquired in both the supine and seated positions using the following parameters: 3D gradient-echo-based T1-weighted image sequence, repetition time (TR) = 18 ms, echo time (TE) = 6 ms, flip angle = 30°, slice thickness = 0.489 mm with no interslice gap, acquisition matrix = 252 × 252, reconstructed matrix = 512 × 512, and in-plane phase-encoding direction set to row. The images were acquired with isotropic voxel spacing (0.489 mm) to allow precise spatial comparison between the postural conditions. Images were acquired in three orthogonal orientations: the axial and sagittal planes. Third, T2-weighted images were acquired in both the supine and seated positions using the following parameters: a three-dimensional gradient-echo-based HYCE (hybrid contrast enhancement) sequence, TR = 10 ms, TE = 5 ms, flip angle = 60°, slice thickness = 0.488 mm with no interslice gap, acquisition matrix = 252 × 252, reconstructed matrix = 512 × 512, and in-plane phase-encoding direction set to row. The images were acquired with isotropic voxel spacing (0.488 mm) to allow precise spatial comparison between the postural conditions. Images were acquired in three orthogonal orientations: axial, sagittal, and coronal. For subsequent quantitative analysis and anatomical delineation, only T1-weighted images were used as they provided a clearer visualization of anatomical structures. The study protocol is illustrated in Figure 1.

### 2.2. Image Preprocessing and Region of Interests-Based Quantitative Analysis

All MRI images acquired under different postural conditions were spatially registered into the same anatomical space using SPM12 (Wellcome Centre for Human Neuroimaging, London, UK), which operates in MATLAB (R2023a; The MathWorks Inc., Natick, MA, USA). Supine images were used as references, and the seated images were co-registered with the corresponding supine images to enable voxel-wise comparisons.

**Figure 2:**
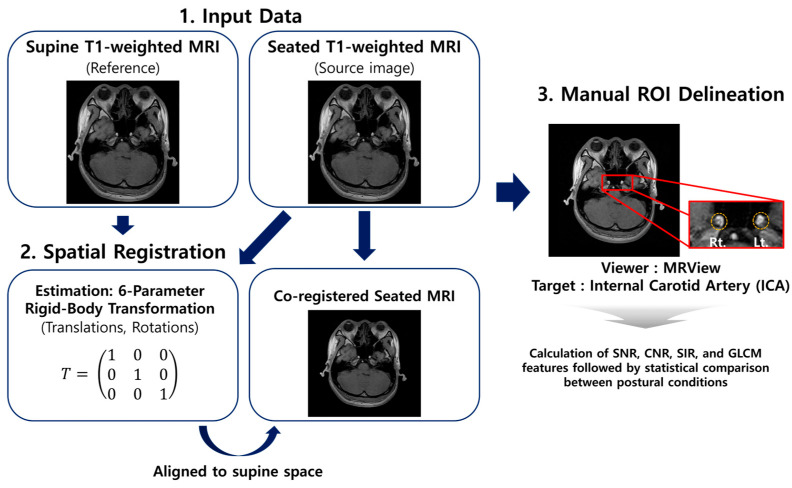
Illustration of the spatial registration workflow and bilateral internal carotid artery (ICA) region of interest (ROI) delineation. Enlarged panels highlight the delineated ICA ROIs on the co-registered seated magnetic resonance imaging. Rt. and Lt. indicate the right and left sides, respectively.

The ICA was designated as the primary region of interest (ROI) using MRView program (version 3.0.3) because of its physiological relevance to hydrostatic pressure gradients and cerebrovascular autoregulation [3]. The ICA was manually delineated on co-registered T1-weighted images by an experienced radiological technician. Figure 2 illustrates the overall analysis workflow, including ICA ROI delineation, extraction of quantitative image features (SNR, CNR, and GLCM metrics), and statistical analysis used to assess the posture-dependent differences between supine and seated conditions.

Quantitative image analysis was performed by calculating the SNR, CNR, and signal intensity ratio (SIR) between the ICA and reference region. SNR, CNR, and SIR are defined as follows:

(1) $$SNR=\frac{S_{ICA}}{\sigma_{noise}}$$

(2) $$CNR=\frac{\left|S_{ICA}-S_{air}\right|}{\sigma_{noise}}$$

(3) $$SIR=\frac{S_{ICA}}{S_{air}}$$

where $S_{ICA}$ represents the mean signal intensity within ICA, $S_{air}$ represents the mean signal intensity measured from an air region located adjacent to the ICA outside the cranial boundary, and $\sigma_{noise}$ depicts the standard deviation of signal intensity measured within this same region.

In addition to intensity-based metrics, a GLCM-based texture analysis was conducted to evaluate the spatial distribution and structural characteristics of the ICA signals associated with postural changes. Textural features, including contrast, correlation, energy, and homogeneity, were extracted from the ICA to quantify subtle structural variations in the vascular signal patterns. The GLCM $P\left(i,j\right)$ represents the probability of the occurrence of two gray levels i and j separated by a defined spatial relationship within the ROI. The following texture features were calculated using the GLCM:

(4) $$GLCM_{Contrast}=\underset{i,j}{\sum }{\left|i-j\right|}^{2}p\left(i,j\right),$$

(5) $$GLCM_{Correlation}=\underset{i,j}{\sum }\frac{\left(i-\mu_{i}\right)\left(j-\mu_{j}\right)p\left(i,j\right)}{\sigma_{i}\sigma_{j}},$$

(6) $$GLCM_{Energy}=\underset{i,j}{\sum }p{\left(i,j\right)}^{2},$$

(7) $$GLCM_{Homogeneity}=\underset{i,j}{\sum }\frac{p\left(i,j\right)}{1+\left|i-j\right|},$$

where $p\left(i,j\right)$ is the probability value in the GLCM for the occurrence of gray levels i and j at a given spatial offset. $\mu_{i}$ and $\mu_{j}$ represent the mean values of the row and column indices of the GLCM, respectively, and $\sigma_{i}$ and $\sigma_{j}$ denote the corresponding standard deviations. The $GLCM_{Contrast}$ measures the degree of local intensity variation, reflecting how sharply the signal intensity changes within the ICA region. Lower contrast values indicated smoother and more uniform signal patterns. The $GLCM_{Correlation}$ evaluates the linear dependency between neighboring pixel intensities, indicating the degree of structural similarity in the vascular signal distribution. The $GLCM_{Energy}$ represents the uniformity of the intensity patterns and increases when the texture becomes more homogeneous. The $GLCM_{Homogeneity}$ measures the similarity of adjacent pixel intensities, with higher values indicating smoother and more consistent ICA signal structures.

### 2.3. Statistical Analysis

All statistical analyses were performed using the MATLAB software (R2023a; MathWorks Inc.). To evaluate the differences in the quantitative image features obtained from the same participants under the two postural conditions, the Wilcoxon signed-rank test, a non-parametric test suitable for paired measurements, was applied. A significance threshold of *p* < 0.05 was applied for all statistical comparisons.

## 3. Results

This study evaluated posture-dependent changes in the vascular signal characteristics of the ICA by comparing low-field MRI acquisitions obtained in the supine and seated positions. Quantitative analyses were performed on the ICA ROIs derived from the co-registered T1-weighted images, and nonparametric paired statistical comparisons were conducted using the Wilcoxon signed-rank test. Significant differences were observed in several signal intensity-related metrics between postural conditions. The seated position showed higher SNR, CNR, and SIR compared with the supine position (median SNR: 17.11 vs. 13.48; median CNR: 21.94 vs. 18.36; median SIR: 10.84 vs. 9.54; all *p* = 0.004). The relative increases in SNR and CNR observed in the seated position indicated reduced noise within the ICA signal and improved discrimination from the surrounding tissues. A higher SNR reflects the stability of the signal amplitude relative to the background, whereas an increased CNR suggests enhanced differentiation between the ICA signal and adjacent anatomical structures. These findings indicate enhanced ICA signal detectability in the seated position relative to that in the supine position. In addition, significant differences in SIR suggest the presence of posture-related variations in ICA signals, potentially reflecting underlying changes in blood flow characteristics associated with postural changes.

The contrast metric in GLCM exhibited a significant decrease in the seated position compared with the supine position (median: 62.01 vs. 145.92, *p* = 0.004), suggesting that posture-related changes differentially affected signal intensity and relative contrast characteristics. First, the contrast reflects the intensity difference between a given pixel and its adjacent pixels within an image. A higher contrast index indicates greater variability in the pixel intensities and the frequent presence of distinct signal levels, reflecting a more pronounced and heterogeneous texture. By contrast, a lower contrast index indicates smaller intensity differences between adjacent pixels, which correspond to a smoother and more uniform texture. In this study, the contrast was significantly lower in the seated position than in the supine position, suggesting that the ICA signal exhibited relatively smoother and more homogeneous textural characteristics in the seated postures. Correlation reflects the linear dependency between pixel intensities within an image, with higher values indicating that pixels follow a more consistent pattern and exhibit stronger inter-pixel relationships. In the present study, the correlation showed a slight increase in the seated position. Energy represents the regularity and uniformity of image texture, with higher values indicating more repetitive and structurally consistent signal patterns. In this study, energy demonstrated a slightly decreasing trend in the seated position, suggesting a modest reduction in textural regularity. Homogeneity describes the similarity between pixel intensities and reflects the overall uniformity of an image. The observed increase in homogeneity in the seated position suggests that the ICA signal exhibited a more uniform distribution. However, the correlation, energy and homogeneity did not show statistically significant differences, indicating the magnitude of postural variation.

Overall, the results show that body posture significantly influences the ICA signal characteristics on low-field MRI. While seated positioning was associated with increased SNR-, CNR-, and SIR-related metrics, the texture features remained largely unchanged between the postural conditions, except for contrast. The individual participant results, along with the group mean and standard deviation for each quantitative metric in the supine and seated positions, and the results of the Wilcoxon signed-rank test, are summarized in Table 1. These results suggest that postural changes primarily influence signal intensity-related metrics (SNR, CNR, and SIR) were significantly higher in the seated position than in the supine position. GLCM contrast was significantly lower in the seated position, indicating a more homogeneous vascular signal pattern. Other textural features, including correlation, energy, and homogeneity, did not show significant postural differences.

## 4. Discussion

This study evaluated posture-dependent vascular signal changes in the ICA by comparing T1-weighted images acquired in supine and seated positions using a 0.25 T low-field MRI system. Signal intensity and texture features were analyzed to characterize the vascular signal properties under different postural conditions. To ensure an accurate voxel-level comparison between postures, all MRI datasets were spatially co-registered using a rigid-body transformation. The anatomical location of the ICA was consistently identified in both supine and seated images with guidance from an experienced radiologist. ROI were delineated within the ICA to enable reproducible signal extraction across postural conditions within the same participants.

Quantitative analysis based on the extracted ROIs revealed significant posture-dependent differences in the ICA signal characteristics, with the seated position demonstrating a higher SNR, CNR, and SIR than the supine position, indicating increased signal stability and relatively reduced noise within the vascular region. Among the GLCM features, the contrast was significantly lower in the seated position, suggesting a smoother and more homogeneous signal distribution within the ICA. Taken together, these findings indicate that postural change exert a greater influence on signal intensity-related properties than on spatial texture patterns of the ICA, and suggest that vascular signal characteristics in low-field MRI might be sensitive to posture-related physiological alterations.

The signal difference observed in the ICA between the supine and seated positions can be interpreted as a physiological adaptation of the cerebral hemodynamics induced by gravity [5,14]. Transitioning from a supine to a seated posture alters the cerebral hydrostatic pressure distribution, venous outflow pathways, and intracranial pressure, and these physiological changes influence arterial inflow to the brain [1,14,31]. In addition, increased parasympathetic activity in the supine position affects vascular states [32,33]. Thus, evaluating the ICA in the seated position is particularly meaningful, as it represents an arterial conduit supplying cerebral blood and allows for the assessment of posture-dependent hemodynamic and physiological adaptations. Furthermore, postural changes influence not only systemic hemodynamics but also craniocervical alignment and vascular geometry [18,28,32]. These alterations affect the vascular diameter and flow velocity and can modify the magnetization state in MRI signals, suggesting that vascular signal differences may occur even in the absence of structural vascular changes [34].

Consistent with this, previous physiological studies utilizing seated phase-contrast MRI have well-documented that transitioning to a seated posture induces hydrostatic pressure gradients, leading to a reduction in ICA cross-sectional area and blood flow velocity [3,35,36]. In alignment with these physiological phenomena, our quantitative analysis demonstrated significantly higher SNR and CNR in the seated position. This increase in signal stability can be attributed to the slower blood flow velocity, which likely reduces flow-related signal loss commonly observed in conventional T1-weighted imaging. Ultimately, this confirms that posture-induced alterations in hemodynamics directly influence traditional image features and contrast mechanisms even in 0.25 T low-field MRI environments.

Low-field MRI, which can simultaneously reflect posture-based acquisition and physiological positional changes, has the potential to enhance vascular signal contrast and improve the visualization of vascular structures [15]. This represents an advantage over conventional high-field MRI systems, in which imaging is typically performed in the supine position owing to fixed magnet configurations, and indicates the clinical feasibility of seated MRI as a practical tool for directly assessing gravity-related cerebrovascular adaptation [25,37]. In particular, such approaches may be useful for evaluating conditions in which symptoms are posture dependent, such as orthostatic hypotension, where abnormalities are not evident in the supine position but emerge during the standing posture [15,38]. Accordingly, ICA-based signal and texture analyses enabled the quantification of posture-dependent vascular signal changes and demonstrated their potential for clinical applications. As an arterial pathway reflecting cerebral inflow, the ICA provides physiologically meaningful information, and assessments based on signal intensity and texture can complement the measures of blood distribution and pressure by sensitively capturing functional vascular responses. This approach offers a practical imaging biomarker capable of evaluating vascular adaptation at the stages preceding overt structural changes [39,40].

Despite these strengths, this study has several limitations. First, this study evaluated ICA signal changes indirectly using image-based metrics and did not directly measure the blood flow velocity or volumetric flow. Thus, it is difficult to conclude whether the observed signal alterations fully correspond to the hemodynamic changes. Future studies combing phase-contrast MRI or ultrasound-based flow measurements are required to validate the relationship between imaging-derived signals and actual flow parameters. Second, the relatively small sample size limited the generalizability of the findings, and inter-individual physiological variability may have influenced the signal metrics. A larger sample size is required to confirm the reproducibility of posture-dependent vascular signal changes. Third, the ROI-based approaches depend on the segmentation accuracy and ROI placement, introducing potential observer dependency. Thus, future studies should extend the analysis to multiple vascular territories beyond the ICA to characterize posture-dependent vascular responses more comprehensively. Fourth, this study exclusively relied on T1-weighted images to evaluate the anatomical structures and signal characteristics. Because T2-weighted imaging can provide complementary information regarding tissue contrast and fluid dynamics, future studies should also incorporate T2-weighted sequences to achieve a more comprehensive evaluation. Finally, the texture and signal intensity changes observed in low-field MRI may arise from the combined effects of blood flow, magnetization physics, and tissue microstructure and thus should not be interpreted as reflecting a single underlying mechanism. Future investigations incorporating multi-sequence and multi-modal approaches are warranted to enable a more precise mechanistic interpretation.

## 5. Conclusions

This study demonstrated that the ICA signal characteristics exhibited significant posture-dependent changes when comparing supine and seated T1-weighted images acquired using a 0.25 T low-field system. Specifically, our quantitative analysis revealed that the seated position yielded significantly higher signal-to-noise ratio (SNR), contrast-to-noise ratio (CNR), and signal intensity ratio (SIR) compared to the supine position, alongside a reduction in GLCM texture contrast. These specific findings indicate a more stable and homogeneous intravascular signal distribution during postural transition. Consequently, this demonstrates that low-field MRI can effectively capture physiological cerebrovascular adaptations to gravity, highlighting the ICA as a sensitive arterial target for evaluating posture-related vascular responses.

The ability of low-field MRI to incorporate posture-based acquisition provides a unique advantage over conventional high-field systems, supporting its potential as a practical imaging approach for assessing functional vascular adaptation and posture-dependent cerebrovascular conditions. Therefore, ICA signal and texture analyses may serve as quantitative imaging biomarkers capable of detecting vascular alterations that precede overt structural changes.

## Figures and Tables

**Table 1:** Quantitative comparison of internal carotid artery (ICA) signal metrics between the supine and seated positions. Individual participant values and group mean ± standard deviation for signal intensity–related metrics and gray-level co-occurrence matrix-based texture features measured within the ICA are presented.

Subject No.	Position	Index of Metric						
		SNR	CNR	SIR	Contrast	Correlation	Energy	Homogeneity
Subject 1	Supine	13.382	4.221	1.207	265.522	0.211	0.081	0.480
	Seated	15.566	17.230	5.127	34.480	0.425	0.062	0.409
Subject 2	Supine	18.482	23.719	7.893	120.986	0.323	0.062	0.310
	Seated	23.546	28.640	9.751	59.439	0.311	0.057	0.313
Subject 3	Supine	11.016	15.846	13.363	145.924	0.388	0.087	0.283
	Seated	21.433	26.261	14.069	21.545	0.146	0.115	0.400
Subject 4	Supine	13.846	19.235	11.272	117.888	0.194	0.038	0.276
	Seated	17.648	22.981	13.808	62.006	0.362	0.036	0.314
Subject 5	Supine	12.368	17.281	9.088	150.270	0.284	0.034	0.250
	Seated	18.259	23.176	13.314	60.262	0.400	0.039	0.368
Subject 6	Supine	14.250	19.037	11.895	146.125	0.344	0.057	0.294
	Seated	15.732	20.283	14.099	115.614	0.476	0.052	0.293
Subject 7	Supine	10.549	15.698	15.038	91.403	0.386	0.024	0.277
	Seated	11.693	16.840	14.836	78.030	0.419	0.024	0.283
Subject 8	Supine	13.519	18.670	12.511	155.011	0.044	0.038	0.293
	Seated	15.684	20.757	12.802	110.383	0.287	0.034	0.283
Subject 9	Supine	13.482	18.357	8.987	111.575	0.273	0.042	0.281
	Seated	17.113	21.936	9.978	81.009	0.317	0.039	0.299
Mean of supine ± SD		13.433 ± 2.281	16.896 ± 5.306	10.139 ± 1.057	144.967 ± 49.947	0.272 ± 0.110	0.051 ± 0.022	0.305 ± 0.068
Mean of seated ± SD		17.408 ± 3.474	22.012 ± 3.848	11.976 ± 3.137	69.196 ± 31.167	0.349 ± 0.098	0.051 ± 0.098	0.329 ± 0.050
Results of Wilcoxon signed-rank test		*W* = 45 *p* = 0.004	*W* = 0 *p* = 0.004	*W* = 0 *p* = 0.004	*W* = 45 *p* = 0.004	*W* = 9 *p* = 0.129	*W* = 30 *p* = 0.426	*W* = 12 *p* = 0.250

SNR, signal-to-noise ratio; CNR, contrast-to-noise ratio; SIR, signal intensity ratio.

## Data Availability

The original contributions presented in this study are included in the article. Further inquiries can be directed to the corresponding authors.

## Abbreviations

CNR	contrast-to-noise ratio
CSF	cerebrospinal fluid
ICA	internal carotid artery
MRI	magnetic resonance imaging
ROI	region of interest
SIR	signal intensity ratio
SNR	signal-to-noise ratio
